# Multi-Response Optimization of Granaticinic Acid Production by Endophytic *Streptomyces thermoviolaceus* NT1, Using Response Surface Methodology

**DOI:** 10.3390/bioengineering3030019

**Published:** 2016-07-27

**Authors:** Sudipta Roy, Suman Kumar Halder, Debdulal Banerjee

**Affiliations:** 1 Microbiology and Microbial Biotechnology Laboratory, Department of Botany and Forestry, Vidyasagar University, Midnapore 721102, India; sry.5@rediffmail.com; 2 PG Department of Biotechnology, Oriental Institute of Science and Technology, Midnapore 721102, India; 3 Department of Microbiology, Vidyasagar University, Midnapore 721102, India; sumanmic@mail.vidyasagar.ac.in

**Keywords:** *Streptomyces thermoviolaceus*, granaticinic acid, antimicrobial, optimization, response surface methodology, Box–Behnken design

## Abstract

*Streptomyces thermoviolaceus* NT1, an endophytic isolate, was studied for optimization of granaticinic acid production. It is an antimicrobial metabolite active against even drug resistant bacteria. Different media, optimum glucose concentration, initial media pH, incubation temperature, incubation period, and inoculum size were among the selected parameters optimized in the one-variable-at-a-time (OVAT) approach, where glucose concentration, pH, and temperature were found to play a critical role in antibiotic production by this strain. Finally, the Box–Behnken experimental design (BBD) was employed with three key factors (selected after OVAT studies) for response surface methodological (RSM) analysis of this optimization study.RSM analysis revealed a multifactorial combination; glucose 0.38%, pH 7.02, and temperature 36.53 °C as the optimum conditions for maximum antimicrobial yield. Experimental verification of model analysis led to 3.30-fold (61.35 mg/L as compared to 18.64 mg/L produced in un-optimized condition) enhanced granaticinic acid production in ISP2 medium with 5% inoculum and a suitable incubation period of 10 days. So, the conjugated optimization study for maximum antibiotic production from *Streptomyces thermoviolaceus* NT1 was found to result in significantly higher yield, which might be exploited in industrial applications.

## 1. Introduction

Microbial secondary metabolites are extremely species-specific, and sometimes the same species isolated from different ecological statuses produce significant variations in secondary metabolite profile [1]. Microbial cell factories are influenced by physical and nutritional parameters to generate subtle-to-significant variation in biosynthetic pathways that originate precious compounds for mankind [2,3]. Most of such compounds have unique structural features and possess versatile bioactivities. Often, such compounds are accumulated in substantial amounts, which is a key factor in their industrial interest. In biotechnological applications, nevertheless, microbial metabolites used as antimicrobials still continue to represent important research and development of new therapeutic agents [4]. The evaluation and optimization of potential, lesser-known, novel microbial bioactive compounds are of increasing interest to the industries [2]. Among bacteria, the order *Actinomycetales* has proved its excellence in the production of a diverse range of secondary metabolites, especially with antimicrobial properties with extensive industrial interests [5]. Specifically, the *Streptomyces* are outstanding in the production of large numbers of antibiotics and other bioactive compounds [6]. 

*Streptomyces thermoviolaceus* is known to produce granaticins [7], a pigmented antibiotic. Granaticins, produced by many *Streptomyces*, are members of a broader class of pyranonapthoquinone microbial metabolites [8] and are generally produced as a mixture of different derivatives (granaticin A, granaticin B, dihydrogranaticin, granaticinic acid) in bacterial fermented broth [9], but *Streptomyces thermoviolaceus* NT1 was found to produce solely granaticinic acid in culture filtrate [10]. Granaticins are reported for antibacterial, antitumor and cytotoxicity against KB cells [11], but granaticinic acid was found to be non-mutagenic and to have strong antibacterial activity against both gram-positive and gram-negative bacteria, including the deadly *Pseudomonas aeruginosa*. It is also potentially inhibitory against MRSA (methicillin-resistant *S. aureus*) and PRSA (penicillin-resistant *S. aureus*) strains [10]. This endophytic strain NT1 certainly draws biotechnological importance for the production of future antibiotics which have a potential anti-infective property, even against resistant bacteria, and due to its production of a single granaticin derivative in fermented liquor.

The present communication explains the enhanced yield of granaticinic acid obtained after thoroughly examining different influencing parameters from single and multidisciplinary approach after significant statistical analysis.

## 2. Experimental Section

### 2.1. Microbial Strains, Maintenance, and Chemicals

The endophytic *Streptomyces thermoviolaceus* NT1 (Gen Bank Acc. No. KJ486841) was taken for this study. The strain is one of our isolated endophytic actinomycetes strains, which was reported for granaticinic acid production [10]. The strain is maintained in ISP2 broth with 30% glycerol at −20 °C. For the assessment of maximum granaticinic acid production, two pathogenic strains were selected; *Bacillus cereus* (ATCC 14579) and *Escherichia coli* (clinical isolate) as test pathogens. The pathogenic strains were maintained in tryptic soy slant culture at 4 °C. All the media components were of analytical grade and procured from HiMedia Laboratories Pvt Ltd. (Mumbai, India).

### 2.2. Inoculum Preparation for Fermentation

StrainNT1 was transferred from stock culture on solid ISP2 medium. It was then incubated at 35 °C for 7 days. Agar blocks containing full-grown strain were cut (1 cm^2^ surface area) and transferred in Erlenmeyer flask (150 mL) containing 30 mL ISP2 liquid medium. The strain was allowed to further grow for 5 days with shaking (150 rpm) at the same conditions up to biomass generation (dry weight) of 50 mg/100 mL medium. This culture was used as inoculum at different optimization steps. 

### 2.3. Production of Granaticinic Acid

Production of granaticinic acid was carried out in 250 mL Erlenmeyer flasks. The endophytic *S*. *thermoviolaceus* NT1 was cultured in 50 mL ISP2 medium having pH 7.0 at 28 °C inside a rotary shaker incubator at 150 rpm shaking speed for 12 days. Unless otherwise specified, during optimization, culture conditions and media compositions were used as optimized in theprevious experimental step.

### 2.4. OVAT Optimization for Production of Granaticinic Acid

Following parameters were optimized by one-variable-at-a-time (OVAT) to determine granaticinic acid production from this strain at increased levels. Production media, initial media pH, incubation temperature, incubation period, inoculum size, and glucose concentration were selected parameters that were analyzed for maximum granaticinic acid production in optimum conditions.

### 2.5. Optimization of Media

Few well-known formulated media have been tested to find the most suitable one for yielding maximum granaticinic acid. For this purpose, the liquid media used were as follows; ISP5, ISP2, SCN, TYG and MS broth. Initial pH of all media was adjusted at 7.0. Fermentation was initiated with 2% inoculum in each 50 mL media inside an Erlenmeyer flask (250 mL). Incubation was carried out at 35 °C for 12 days with shaking at 150 rpm. Cell-free culture broth was analyzed for antimicrobial activity.

### 2.6. Determination of Optimum Glucose Concentration

Optimum glucose concentration for maximum antimicrobial activity was also determined. For this, ISP2 media (devoid of glucose) was taken in separate Erlenmeyer flasks and ameliorated with different glucose concentrations (g%) (0, 0.2, 0.4, 0.6, 0.8, and 1 g%). Each fermentation media was inoculated with 5% (*v/v*) seed culture of NT1. Production was carried out at pH 7.0, 35 °C, for 12 days with shaking at 150 rpm. After completion of fermentation, culture filtrates of each media were evaluated for antimicrobial activity.

### 2.7. Determination of Initial Media pH 

The endophytic strain NT1 was fermented in a series of individual ISP2 media of different pH (4, 6, 7, 8, and 10) at 35 °C. Fermentations were carried out for up to 12 days. Culture filtrates were analyzed. 

### 2.8. Determination of Incubation Temperature 

The endophytic strain NT1 was fermented in a series of individual ISP2 media adjusted to pH 7.0 under different incubation temperatures (20, 30, 35, 40 and 45 °C). Fermentations were stopped at the 12th day and culture filtrates were analyzed.

### 2.9. Determination of Fermentation Period 

Liquid fermentation was allowed in ISP2 media with pH 7.0 at 35 °C for fifteen successive days with shaking at 150 rpm. Samples were taken out at each day. Antimicrobial activity as well as dry cellular weight was recorded each day. 

### 2.10. Determination of Most Suitable Inoculum Size

Suitable inoculum size plays a critical role in antibiotic production by *Streptomyces* [12]. Optimum inoculum concentration was determined after transferring different inoculum size (0.5%, 1%, 2%, 5%, and 10%; *v/v*) in ISP2 media, and production was carried at pre-optimized conditions. 

### 2.11. Optimization of Granaticinic Acid Production Using Box–Behnken Design

A more applicable optimization for antibacterial production by this strain was performed with response surface methodology (RSM). The Box–Behnken experimental design (BBD) was employed with three key factors selected from OVAT studies. The experimental design was conducted with the three most influencing independent factors, each at three different levels (−1.0, 0, and +1.0), encompassing a total of 17 experiments (Table 1). Antibacterial production was evaluated, adopting a quadratic polynomial Equation:

*Y* = β_0_ + ∑ β_i_χ_i_ + ∑ β_ii_χ_i_^2^ + ∑ β_ij_χ_i_χ_j_

The equation implies that the input variables χ_i_ and χ_j_ influence the predicted response *Y*. β_0_ is the intercept of the regression equation, β_i_ is the linear coefficient, β_ii_ is the quadratic coefficient, and β_ij_ is the interaction coefficient. A functional model was generated by the multiple regression analysis of the responses, and its efficiency was verified by ANOVA and Fisher’s F-test. The mutual interactions among independent variables were represented by three-dimensional response surface plots.

### 2.12. Validation of RSM Predictions

Strain *S. thermoviolaceus* NT1 was inoculated (5%) in three separate Erlenmeyer flasks (250 mL) containing 50 mL ISP media. Initial media pH, incubation temperature, and glucose concentration was maintained as predicted optimum by the RSM study. Fermentation was carried out for 10 successive days, and antimicrobial activity of culture filtrates against *B. cereus* (ATCC 14579) was determined on MHA media by agar well diffusion assay in three replicates [13]. Inhibition zone produced was compared with RSM predictions.

### 2.13. Statistical Analysis

All experiments were carried out in triplicate, and data are represented here as mean ± SEM. The RSM experimental design and concomitant regression analysis of experimental data were executed using Design-expert 8.0software (STAT-EASE Inc., Minneapolis, MN, USA). The response surface curves, as well as subsequent contour plots from the results of RSM experiment, were also drawn with the same software. The OVAT data analysis was performed using Microsoft Excel 2007 software. 

### 2.14. Estimation of Granaticinic Acid at Optimization Phases

The relative amount of granaticinic acid produced was determined after comparing zone of inhibitions produced by different concentrations of 2-(4-Hydroxy-7-methoxy-5,8-dioxo-3,4,5,8-tetrahydro-1H-isochromen-3-yl)-acetic acid ethyl ester (similar class to the antimicrobial compound pyranonapthoquinone [14]) and granaticinic acid at different phases of optimization.

Concentration of granaticinic acid in culture filtrates was determined as amount of granaticinic acid (volume of culture filtrate) loaded into wells of MHA agar plates. 

Volume of well was calculated from the equation:

V = πr^2^h, where r = radius and h = height of well = (π) (0.25)^2^0.5 [well diameter = 0.5 cm and height = 0.5 cm]

## 3. Results

To make the production of the antibiotic feasible, it is necessary to develop the optimum production conditions, as product yield greatly varies between pre- and post-optimized conditions [15]. The strain *Streptomyces thermoviolaceus* NT1 was previously reported for production of granaticinic acid, a broad spectrum pyranonapthoquinone antibiotic. 

### 3.1. OVAT Optimization of Granaticinic Acid Production

**Figure 1:**
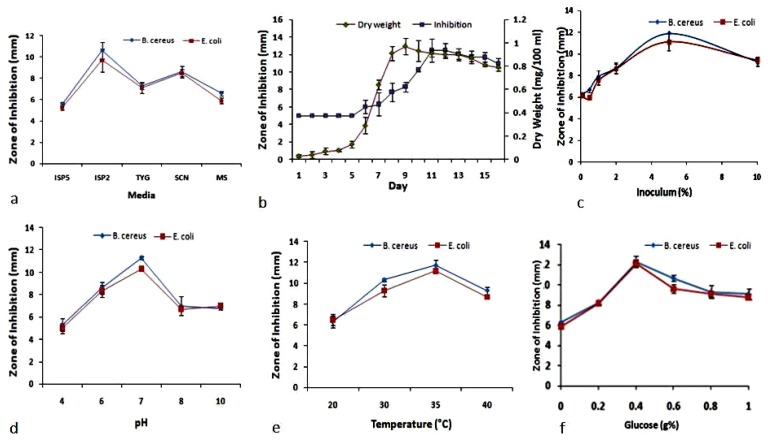
Effect of different influencing parameters in granaticinic acid production (antimicrobial activity) by one-variable-at-a-time (OVAT) study; (**a**) influence of media on activity; (**b**) growth curve vs. antimicrobial production; (**c**) influence of inoculum concentration; (**d**) influence of initial media pH; (**e**) influence of incubation temperature; (**f**) influence of glucose concentration on antimicrobial production.

The endophytic *S. thermoviolaceus* NT1 was fermented in an Erlenmeyer flask for the production of granaticinic acid. Its growth with granaticinic acid production and other optimization results are presented in Figure 1. An increase in the zone of inhibition always indicates an increased yield. It was observed that granaticinic acid production initiates at the 5th day of incubation, after which it exponentially increased and reached its highest level at the 10th day. Biomass generation was also noticed exponentially after a 4 day lag phase. After 8 days of incubation, the organism entered in stationary phase, while antimicrobial production was markedly increased after that (Figure 1b). ISP2 media was found to be the most-preferred production media, and considerable production was also achieved in SCN broth (Figure 1a). Highest antimicrobial production was observed with 5% seed (Figure 1c) as starter of the said fermentation. Study of the relationship between granaticinic acid production with initial media pH and incubation temperature revealed that pH 7.0 and 35 °C temperature is the optimum condition for maximum antimicrobial yield (Figure 1d,e respectively). Initial media pH lower than 6 (or slightly alkaline environment) is not at all suitable for granaticinic acid production by this strain. There was a sharp relationship between glucose concentration and granaticinic acid production. It was noticed that 0.4% glucose is the most effective concentration (Figure 1f) for antimicrobial production by *S*. *thermoviolaceus* NT1. Almost similar antimicrobial activity was observed against the gram-positive *B. cereus* and the gram-negative *E. coli* during production optimization through OVAT fashion (Figure 1).

### 3.2. Box–Behnken Optimization for Granaticinic Acid Production

**Figure 2:**
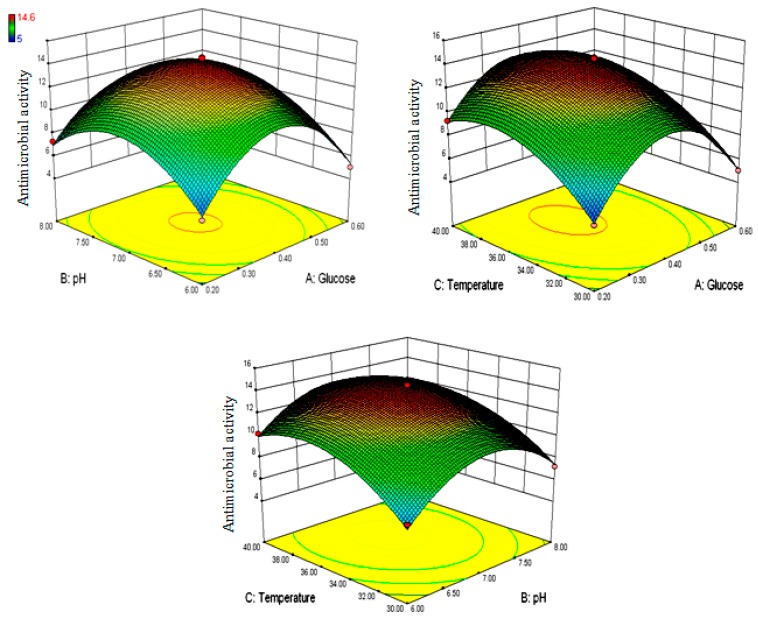
The 3D plot and 2D projections showing most important interactions for maximum antimicrobial production by TNT1, a; between glucose concentration (g%) and initial media pH at temperature 35 °C; b: glucose concentration (g%) and temperature (°C) at initial media pH 7.0; c: between temperature (°C) and initial media pH 7 at glucose concentration 0.4 (g%)

The conventional OVAT approach faces some restrictions, especially when multiple significantly influencing parameters work together. So, a conjugated OVAT–RSM approach is always preferable [16]. In this optimization experiment, a three level Box–Behnken design of three factors (initial pH of the fermentation medium, incubation temperature during fermentation, and glucose concentration) with five replicates at the center point of each factor was implicated (Table 1) as the model for the analysis of granaticinic acid production. Experimental design, together with predicted and measured responses of antibacterial production, is presented in Table 1. Significant variations in antibacterial production by both strains were observed at different production conditions, whereas the center point condition was found to always yield maximum antibacterial production compared to others. Multiple regression analysis of predicted response Y for antibacterial production by individual strain was described as:

Y*_Granaticinate_* = 14.24 − 0.625A + 0.1875B + 1.4625C − 0.5AB − 0.45AC − 0.375BC − 5.1075A^2^ − 3.4825B^2^ − 2.4325C^2^

where Y*_Granaticinate_* implies yield of granaticinc acid by strain NT1, and A, B, C are coded factors of glucose concentration (g%), initial media pH, and temperature, respectively. The goodness of fit of the conducted RSM was evaluated with the experimental outputs by performing the regression study (Table 2). According to the constructed model, three-dimensional response surface plots (Figure 2) were generated by Design Expert 8.0 software to better understand the effects of the variables and their interactions on the yield of granaticinic acid. The 3D plots (Figure 2) showing interactions between the selected variables aided rapid evaluation of the critical values of each variable for maximum production. It was observed that all of the quadratic surfaces, rather than linear planes, were significant in the 3D plots.

### 3.3. Validation Study

**Figure 3:**
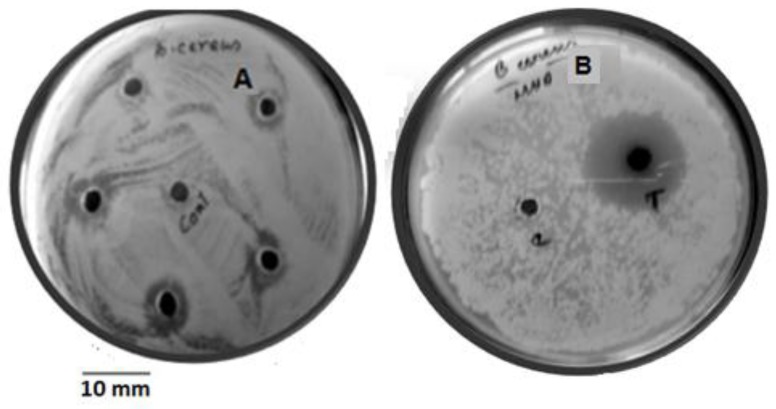
Growth inhibition of *B. cereus* by granaticinic acid at different stages of optimization; (**A**) un-optimized; (**B**) RSM optimized.

Validation of these predictions related to granaticinic acid production by this endophytic *Streptomyces* was assessed by laboratory trials in Erlenmeyer flask culture. Antimicrobial activity of culture filtrates grown in RSM optimized conditions were compared (against *B. cereus* (ATCC 14579)) with the predicted stipulation of the RSM study. Increased antimicrobial activity was observed at each stage of optimization (Figure 3).

### 3.4. Yield of Granaticinic Acid

**Figure 4:**
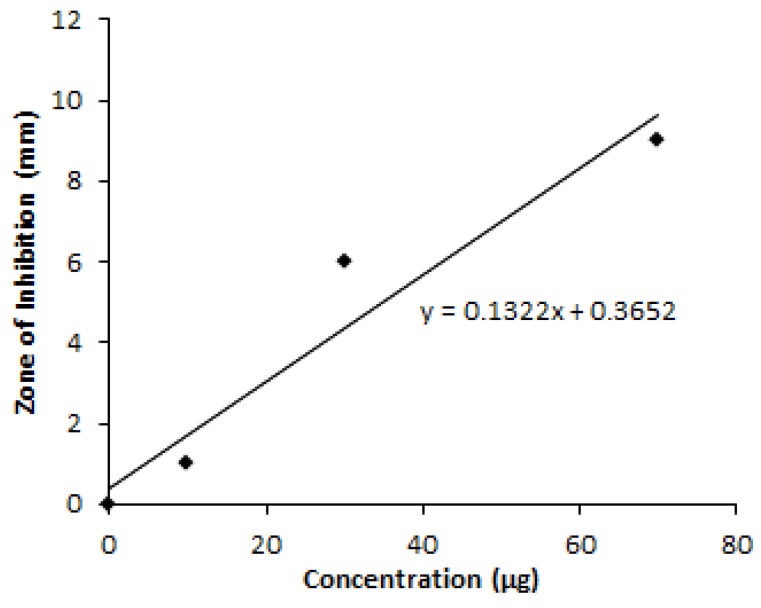
Standard curve of pyranonapthoquinone antimicrobial to determine granaticinic acid yield.

Culture filtrates of NT1 produced at different phases of optimization of granaticinic acid production were compared with standard data provided by a similar compound synthesized by Lagorio, et al., (2006). From the standard curve (Figure 4), the most probable presence of granaticinic acid in culture filtrates are presented in Table 3. Antibiotic production increased by up to 3.30-fold, which is equivalent to almost 61.35 mg/L, when the three most significant influencing parameters worked cooperatively at their critical value. 

## 4. Discussion

In this study, optimization of culture conditions for the production of granaticinic acid from endophytic strain *S. thermoviolaceus* NT1 has been studied through OVAT, factorial design, and RSM analysis. The production of secondary metabolites with antimicrobial property is a common phenomenon among the genus *Streptomyces*. This group of bacteria has been isolated so much from traditional ecotopes like water or soil that isolation of *Streptomyces* with unusual secondary metabolites profile has become rare [17]—the quest for endophytic microorganisms indicates some new leads. The endophytic *S. thermoviolaceus* NT1 produces granaticinic acid, which is considered to be significantly pharmaceutically important due to its anti-*Pseudomonas* properties and effectiveness against resistant pathogens. It is well known that the production of antibiotics varies substantially from strains, species, and certainly with the cultural conditions. Growth temperature, media pH, media compositions, limiting substrate concentration, and obviously incubation time influence the microbial growth, which might have effects on onset and intensity of secondary metabolisms [18]. In this experiment, the approach successfully allowed the determination of critical combinatorial values of the most influencing parameters that give the highest antibiotic yield. Any change in the yield of antimicrobial production during OVAT or RSM optimization was determined by measuring zone of inhibition produced by culture filtrates. ISP2 media, which was found to be the most suitable antibiotic production media [19,20], contains malt extract, which consists of various malt hydrolyzed products as carbon source and yeast extract provides an organic nitrogen source and vitamin precursors to facilitate microbial growth. Aside from that it contains glucose as the principal carbon source for growth. The other media employed for granaticinic acid production in this experiment did not support its production by the strain; it might be that the nutritional components were not favorable. Less production was observed in SCN broth, indicating that organic carbon and nitrogen played critical role in the production of active compound. In OVAT optimization, the critical concentration of glucose was found to be 0.4 g% for maximum antimicrobial yield. A high glucose concentration always supports higher biomass generation, but secondary metabolites are produced under stressed conditions. In this experiment, it was found that higher glucose concentrations were not satisfactory for this purpose, as it might avoid nutritional stress. It is also assumed that higher levels of glucose may also inhibit some essential enzyme in the biosynthetic pathway of granaticinic acid. Earlier study shows the use of glutamate as sole carbon source for granaticin production from *Streptomyces thermoviolaceus* sub sp. *Thermoviolaceus* NCIB 10076 [21]. This substrate could be used both as nitrogen and carbon sources for cellular demand, and high yield of biomass generation was found with it. Use of glucose in this study avoids the generation of ammoniacal nitrogen that may reduce antibiotic yield, as in the case of *S.* *fradiae* [22]. Besides, glucose easily generates a large amount of acetate, which is the principle building block of granaticinic acid via a polyketide-derived secondary metabolic pathway. The NT1strain showed a maximum granaticinic acid production at 35 °C in the OVAT study. It explained the well-characterized “tropophase” up to 7 days, and then the “idiophase” was initiated when antibiotic concentration sharply reached its highest point. This nature is highly expected in commercial batch production of valuable products, as complete utilization of substrate is achieved [23]. More or less granaticin production is reported at pH 7.0 from all actinomycetes strains [21], which is collinear to our study. 

Nowadays, before going to microbial fermentation, RSM-based optimization is widely accepted due to its higher efficiency, reasonable design for integrated analysis of variants, and mathematical modeling with recommended values of most possible optimum conditions, along with corresponding product yield. The approach is techno-economically more precise and viable than univariate strategies, and more acceptable industrially [24]. In most cases, OVAT followed by RSM is strongly recommended to find out the critical values of those significant factors [16]. The *F* test data was checked, and the model *F*-value of 145.416607 indicated that the models were significant. It was found that there was only a 0.01% chance that an *F*-value this large could occur due to noise. The adjusted determinant coefficient (*R*^2^ADJ) of the polynomial model was measured to test the goodness-of-fit of the regression equation. The value of *R*^2^ADJ was calculated as 0.98783963. It shows that there existed a high degree of correlation between the measured and predicted values, and more than 96% variations in the response could be explained by that second order polynomial equation. Adequate precision measured the signal-to-noise ratio with a desired value of 29.76—i.e., greater than 4—which in turn indicated an adequate signal, and the model could be used to navigate the design space. The lack of fit *F*-value of 0.84 and 0.75 for both the models implies that the lack of fit was not significant relative to the pure error, and hence the fitness of the model was good. The resulted model *p*-value (*p* < 0.0001) shows that the model equation was suitable to explain the response of experiment pertaining to antimicrobial production by the strain NT1. The *p*-value for lack of fit for the equations (0.1556) is significantly higher than 0.05, indicating the high degree of accuracy and consistency of the experimental values. The *p*-value of probability >*F* less than 0.05 also indicated that the model terms were significant. In these models, the linear and the quadratic effects of glucose concentration (g%), initial media pH, and temperature (°C) were significant (*p* < 0.05). The most effective interaction was obtained between initial media pH and glucose concentration (g%, *p* < 0.05).

The 3D model predicted a maximum response of 14.49 mm (zone of inhibition against *B. cereus* ATCC 14579) when the uncoded levels of the three most significant variables for strain NT1 were initial media glucose concentration 0.38 g%, pH 7.02, temperature 36.53 °C within the experimental range. Validation of these predictions related to antimicrobial production was assessed by laboratory trials in Erlenmeyer flask culture with the predicted stipulation and 14.4 ± 0.031 mm (inhibition zones were observed after 10 days of fermentation at optimized conditions against strain ATCC 14579). This finding certainly established a significant correlation between expected and investigational antimicrobial production. Experimental verification established the fitness and validation of the model, and the best possible conditions were finally selected through the least number of experiments compared to any other design.

The relative yield of granaticinic acid (61.53 mg/L) revealed the importance of such statistical multi-response optimization in the commercial production of antibiotics. The high degree of similarity between prediction and experimental output certainly encourages such process optimization in antibiotic production. About 3.30-fold enhanced production in any pharmaceutical industry is no doubt considerable. So, this strain could certainly be expedient to its pharmaceutical development.

## 5. Conclusions

Production of the antimicrobial compound is found to be critically dependent on few culture conditions as well as nutritional status. In this study, any endophytic *S. thermoviolaceus* NT1 is first employed for optimization of antimicrobial production by OVAT followed by RSM using Box–Behnken design. The critical value of three crucial growth parameters (glucose concentration, media pH, and temperature) were determined when interacting associatively in fermentation for the highest yield of granaticinic acid. Such multi-parameter integrated optimization is practically important because during fermentation, microorganisms have to face all influential parameters at same time. Validation experiments for the proposed model were also carried out to verify the adequacy and accuracy of the generated model, and results showed that the predicted value agreed with the experimental values well. The best combination resulted after RSM analysis provided a three-fold increased production of granaticinic acid in 10 days with 5% starter in Erlenmeyer flask culture. The optimum culture conditions achieved in this experiment set the basis for further study with large scale batch fermentation in a fermenter for granaticinic acid production from *S. thermoviolaceus* NT1. 

## Figures and Tables

**Table 1:** Experimental design and results of the Box–Behnken design for optimization of antimicrobial production by *Streptomyces thermoviolaceus* NT1.

Run	Independent Variables			Response (Growth Inhibition, mm)	
	A: Glucose (g%)	B: pH	C: Temperature (°C)	Observed	Predicted
1	0.6	7.0	30	5	5.0625
2	0.2	8.0	35	7.3	6.9625
3	0.2	7.0	30	5.3	5.4125
4	0.4	8.0	30	7.2	7.425
5	0.6	6.0	35	5	5.3375
6	0.2	7.0	40	9.3	9.2375
7	0.4	7.0	35	14.2	14.24
8	0.4	8.0	40	9.2	9.6
9	0.4	7.0	35	13.9	14.24
10	0.4	7.0	35	14.6	14.24
11	0.4	6.0	40	10.2	9.975
12	0.2	6.0	35	5.3	5.5875
13	0.6	8.0	35	5	4.7125
14	0.4	7.0	35	14.5	14.24
15	0.4	6.0	30	6.7	6.3
16	0.4	7.0	35	14	14.24
17	0.6	7.0	40	7.2	7.0875

**Table 2:** ANOVA for response surface quadratic regression model of antimicrobial production by *Streptomyces thermoviolaceus* NT1.

Source	Sum of Squares	df	Mean Square	*F*-Value	*p*-Value Prob>F
**Model**	228.0028529	9	25.33365033	145.416607	<0.0001
**A**	3.125	1	3.125	17.93767938	0.0039
**B**	0.28125	1	0.28125	1.614391144	0.2445
**C**	17.11125	1	17.11125	98.2195572	<0.0001
**AB**	1	1	1	5.740057401	0.0478
**AC**	0.81	1	0.81	4.649446494	0.0680
**BC**	0.5625	1	0.5625	3.228782288	0.1154
**A^2^**	109.8381316	1	109.8381316	630.47718	<0.0001
**B^2^**	51.06444737	1	51.06444737	293.112859	<0.0001
**C^2^**	24.91392105	1	24.91392105	143.0073369	<0.0001
**Residual**	1.2195	7	0.174214286		
**Lack of fit**	0.8475	3	0.2825	3.037634409	0.1556
**Pure error**	0.372	4	0.093		
**Cor total**	229.2223529	16			
**R-Squared**	0.994679838				
**Adj R-Squared**	0.98783963				
**Pred R-Squared**	0.938307718				
**Adeq Precision**	29.76196049				

**Table 3:** Comparative yield of granaticinic acid at different optimization steps.

Sample Tested	Zone of Inhibition (mm)	Amount of Granaticinic Acid (mg/L)	Fold Increased
Crude	8.83	18.64	0
OVAT*	11.73	40.58	2.17
RSM	14.5	61.53	3.30

* Average zone of inhibition was considered in case of OVAT optimization.
